# Good Teacher Guidance, Whence Academic Efficacy? Parallel Mediating Path of Positive and Negative Academic Emotions in Junior High Students

**DOI:** 10.3390/bs16030415

**Published:** 2026-03-12

**Authors:** Wenmei Sun, Daixin He, Binxin Dong, Xubo Liu

**Affiliations:** Faculty of Education, Henan Normal University, Xinxiang 453007, China; 2410283124@stu.htu.edu.cn (D.H.); dongbinxin@stu.htu.edu.cn (B.D.); 19100274046@stu.htu.edu.cn (X.L.)

**Keywords:** perceived teacher support, academic emotions, academic self-efficacy, academic competence self-efficacy, academic behavior self-efficacy

## Abstract

Background: To examine the relationships among perceived teacher support, academic emotions, and academic self-efficacy among junior high school students. Methods: A survey was distributed to 376 junior high students, utilizing scales for perceived teacher support, adolescent academic emotions, and self-efficacy. Data analysis included descriptive, correlation, and linear regression methods in SPSS 27.0, with mediation effects assessed using the PROCESS macro. Results: (1) Students’ perceived teacher support significantly and positively predicted academic self-efficacy (*β* = 0.22, *t* = 6.07, *p* < 0.001). (2) positive academic emotions (*β* = 0.44, *t* = 10.68, *p* < 0.001) and negative academic emotions (*β* = −0.32, *t* = −8.23, *p* < 0.001) independently mediated the relationship between perceived teacher support and students’ academic self-efficacy. (3) The mediating effect of positive academic emotions (effect size = 0.21) between perceived teacher support and academic competence self-efficacy was significantly greater than that of negative academic emotions (effect size = 0.05). However, the mediating effects of positive academic emotions (effect size = 0.11) and negative academic emotions (effect size = 0.06) on the relationship between perceived teacher support and academic behavior self-efficacy did not significantly differ. Conclusions: Perceived teacher support directly enhanced academic self-efficacy both directly and indirectly through academic emotions, with positive academic emotions offering a more effective pathway than negative emotions.

## 1. Introduction

Academic self-efficacy (ASE) is a key component of middle school students’ subject-specific core competencies. In recent years, this concept has been explicitly articulated in major national policy documents. In the Special Action Plan for Comprehensively Strengthening and Improving Student Mental Health Work in the New Era (2023–2025), the cultivation of students’ positive psychological qualities, including self-confidence, self-respect, and optimism, is highlighted as a key developmental goal (The State Council of the People’s Republic of China & Seventeen Departments Including the Ministry of Education, 2023). As a basic psychological process that supports individual agency, ASE not only contributes to academic performance but also underpins students’ mental health development (Wu & Fu, 2024). Accordingly, clarifying how ASE develops and how it can be strengthened is vital for fostering core psychological qualities and for advancing adolescents’ mental health and overall growth.

In this study, ASE is conceptualized as students’ self-perceived capacity to regulate learning activities and apply effective strategies to meet the demands of particular academic tasks (Bian, 2004). Its meaning is typically reflected in two complementary facets. One facet concerns confidence in understanding disciplinary knowledge and resolving learning difficulties. The other facet involves students’ perceived competence in self-regulation and in the effective use of learning strategies. Social Cognitive Theory suggests that learners’ self-appraisals of capability substantially shape their motivation and guide their academic behavior (Bandura, 1997). When efficacy beliefs are strong, students tend to approach learning with adaptive mindsets and sustained effort; when these beliefs are weak, withdrawal and avoidance are more likely. However, accumulated evidence suggests that junior high school students often report moderate-to-low levels of ASE, and these beliefs may be relatively unstable. Evidence from a large cross-sectional survey conducted in western China, for example, indicated that around 35% of students simultaneously reported high academic pressure and high adaptation-related pressure, whereas fewer students expressed strong confidence in attaining their desired academic outcomes (L. Wang et al., 2016). In practice, insufficient academic confidence has become a common concern among junior high school students (Pintrich & De Groot, 1990). This pattern is closely tied to the developmental features of early adolescence, a pivotal transition marked by rapid physical and psychological change. In this time, students must adapt to increasingly demanding curricula, intensified performance pressure, and competitive progression systems (Gao et al., 2024; Hou & Chen, 2016). Against this background, examining the determinants of junior high school students’ ASE and the mechanisms through which they operate is practically urgent for reducing psychological strain and improving learning quality.

Teacher support is widely regarded as a salient environmental condition linked to adolescents’ social adjustment, motivation, and academic attainment (Sakiz et al., 2012). Perceived teacher support (PTS) refers to students’ perceived availability and quality of teachers’ supportive actions (e.g., warmth, care, and differentiated responsiveness), and it represents a salient psychological resource in classroom contexts (Babad, 1990; H. X. Zhu et al., 2024). This provides a crucial social cognitive foundation for students to develop the belief that ‘I can excel in learning.’ From the perspective of social cognitive theory, teachers directly convey the efficacy message of ‘you can succeed’ to students by offering feedback on their abilities and strategic guidance, thereby influencing their self-assessment of competence (Bandura, 1997). This process is further elaborated in Situated Cognition Theory, which posits that an individual’s cognitive evaluation is context-dependent (Brown et al., 1989). ASE is context-sensitive and develops through ongoing interactions within concrete learning environments (Greeno & Middle School Mathematics through Applications Project Group, 1998). Supportive teaching practices can shape task features (e.g., perceived challenge and interaction patterns) and thereby influence students’ moment-to-moment judgments of capability (Wen et al., 2023; X. C. Zhu et al., 2025). In conclusion, the social cognitive theory provides a core framework for understanding the function of “efficacy information transmission” in teacher support, while the situational cognitive theory supplements this process by explaining the specific conditions from the perspective of “situational embeddedness”.

Furthermore, from the perspective of psychological needs fulfillment, the Self-determination Theory provides additional insights into how PTS reinforces ASE. The effectiveness of PTS stems from its alignment with three fundamental psychological needs: belonging needs through care, competence needs through scaffolding guidance, and autonomy needs through choice empowerment (Ryan & Deci, 2020). This perspective offers a deeper psychological mechanism for the “efficacy information transmission” emphasized by social cognitive theory. Specifically, the efficacy information of PTS is effectively received and internalized by students because it simultaneously addresses their basic psychological needs for relationships, competence, and autonomy.

Research also indicates that for students experiencing temporary academic setbacks or adaptation difficulties, teacher support has a more pronounced effect on promoting their self-belief (Mercer et al., 2011). Furthermore, by providing students with appropriate autonomy and trust, teachers can effectively foster their engagement and initiative in learning, thereby strengthening their academic self-confidence (G. Wang & Zhao, 2022). On the basis of the theoretical arguments and empirical evidence reviewed above, Hypothesis 1 is proposed: PTS positively predicts junior high school students’ ASE.

Although the predictive association between PTS and ASE has been documented (Liu et al., 2021), the processes underlying this link remain insufficiently specified. According to Pekrun’s (2006) control-value theory, environmental factors (e.g., PTS) shape students’ academic emotions, which in turn influence their cognitive evaluations of academic performance (e.g., ASE). Academic emotions refer to the emotional experiences evoked during the learning process, such as pleasure, frustration, or boredom encountered while studying, as well as feelings of joy, anxiety, or shame associated with academic performance (Pekrun, 2006). In junior high school, adolescents are also in a sensitive phase of neural maturation, and reorganization within amygdala–prefrontal circuitry is associated with heightened emotional reactivity relative to childhood (Caballero et al., 2016). Consequently, academic emotions constitute a plausible pathway through which ASE may be shaped during this period.

Positive academic emotions (PAE) denote positive affective experiences in academic settings (e.g., enjoyment, hope, and pride) (Pekrun, 2006). The Control-Value Theory provides a core theoretical framework for understanding the mediating role of academic emotions. It posits that teachers can enhance students’ perceived control and value appraisal of learning activities by offering competency support (e.g., growth feedback) and learning support (e.g., scaffolding guidance), thereby promoting PAE or inhibiting NAE (Pekrun, 2006). Empirical accounts suggest that such positive emotions operate through behavioral means. Behaviorally, positive emotions can broaden attentional scope, promote cognitive flexibility and strategic engagement, encourage exploration (e.g., approaching challenging tasks), and increase opportunities for mastery experiences that strengthen confidence (Eysenck et al., 2007). Therefore, Hypothesis 2 is proposed: PTS is expected to predict ASE indirectly via PAE.

Negative academic emotions (NAE) include unpleasant states such as anxiety, boredom, and shame (Pekrun, 2006). According to the control-value theory, when students have less PTS, their sense of academic control diminishes, which may trigger negative emotions such as anxiety and burnout (Pekrun, 2006). Drawing on the stress-buffering model by Cohen and Wilis (1985) provides a complementary explanation for this mechanism: PTS can function as a protective psychological resource that mitigates anxiety and shame-related experiences. Specifically, its mechanism of action can be explained from two dimensions: With respect to academic competence self-efficacy, anxiety occupies working memory resources (Eysenck et al., 2007), weakening students’ judgment of their own capabilities and prompting individuals to attribute failure to insufficient ability. PTS, through empathetic responses and strategic assistance, can reduce cortisol levels and promote adaptive attributions (Casey et al., 2019). In terms of academic behavior self-efficacy, boredom readily triggers avoidance behaviors in learning (such as procrastination), disrupting the positive reinforcement loop of “effort–success” and undermining students’ confidence in their self-management abilities (Jia et al., 2020). Therefore, Hypothesis 3 is proposed: PTS is expected to predict stronger ASE by reducing NAE.

**Figure 1:**
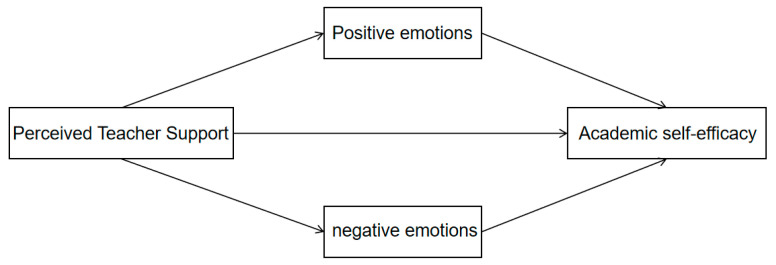
Assumed Model.

Overall, PAE and NAE are conceptually and functionally distinct in definition, roles and cognitive–behavioral consequences (Goetz et al., 2020). While existing studies have separately examined the predictive roles of PTS and academic emotions on ASE, systematic empirical research remains lacking regarding how these two emotional states function as parallel mediators between PTS and ASE. Building upon social cognitive theory and control-value theory, this study constructs a parallel mediation model to investigate the predictive effect of PTS on junior high school students ‘ASE, with particular focus on exploring the parallel mediating role of PAE and NAE in this relationship. The proposed model is illustrated in Figure 1. Through model testing, this research aims to clarify the emotional pathways through which PTS predicts ASE, thereby providing theoretical insights into the formation mechanisms of ASE in junior high school students. Additionally, it offers preliminary empirical evidence to enhance students’ mental health and academic adaptation capabilities.

## 2. Materials and Methods

### 2.1. Participants

The survey was carried out in one junior high school located in Henan Province, China. We handed out 400 paper questionnaires and retained 376 usable cases, corresponding to a 94% valid-return rate (n = 376). Participants were 13.37 years old on average (*SD* = 0.69). The sample consisted of 220 Grade 7 students (58.5%) and 156 Grade 8 students (41.5%). Boys and girls were equally represented (188 each; 50.0% vs. 50.0%). Nearly half of the respondents reported holding a class officer position (n = 187, 49.7%), while the remainder did not (n = 189, 50.3%).

### 2.2. Measures

#### 2.2.1. PTS Questionnaire

All constructs were assessed through self-administered questionnaires. PTS was measured with Ouyang’s (2005) PTS Questionnaire. The instrument covers three domains: learning/academic support, emotional support, and competence support. Items were answered on a 6-point agreement scale from 1 (strongly disagree) to 6 (strongly agree). Reliability in the current dataset was satisfactory (total α = 0.88); the three subscales showed α values of 0.73, 0.80, and 0.75.

#### 2.2.2. Adolescent Academic Emotion Questionnaire

Students’ academic emotions were evaluated using the Adolescent Academic Emotion Questionnaire developed by Dong and Yu (2007). Each item was rated for intensity on a 5-point scale (1 = barely noticeable, 5 = extremely intense). Internal consistency was acceptable for the overall scale (α = 0.82) and was high for the positive- and negative-emotion subscales (α = 0.88 and α = 0.94, respectively).

#### 2.2.3. ASE Scale

Academic self-efficacy was assessed with Liang’s (2002) ASE Scale. The total scale demonstrated adequate reliability (α = 0.84). The ability-related self-efficacy subscale yielded α = 0.90, and the behavior-related self-efficacy subscale yielded α = 0.76.

### 2.3. Statistical Analysis

Responses were coded and analyzed in SPSS 27.0. Possible common-method variance was checked via Harman’s one-factor procedure. Scale reliability was evaluated using internal-consistency coefficients (Cronbach’s α). Independent-samples t tests were then used to examine whether ASE differed across gender, grade and class officers. Mediation was tested with Hayes’ PROCESS macro (Model 4) based on 5000 bootstrap resamples, and Gender, grade and class officers were included as covariates in the mediation model. Indirect effects were treated as significant when the bias-corrected 95% bootstrap confidence interval excluded zero. Statistical significance was set at α = 0.05, and the dataset contained no missing values.

## 3. Results

### 3.1. Common Method Bias

Because all variables were measured via self-report, in order to reduce the common method bias from the source, the corresponding control measures were taken in the research procedure, including anonymous assessment, reverse design of some items, randomization of item order and selection of a mature scale. To further diagnose whether there is a significant common method bias in the data, a Harman single-factor test was conducted. Twenty-six factors had eigenvalues >1, and the first factor explained 20.12% of the variance (<40%). This indicates that the common method bias in this study is within an acceptable range and does not pose a serious threat to the reliability of the research conclusions. It should be noted that the Harman single-factor test is merely a statistical diagnostic tool, and its results primarily serve as supplementary evidence for the absence of significant common method bias, rather than proving that common method bias is “completely absent.”

### 3.2. Descriptive Statistics and Correlations

#### 3.2.1. Demographic Differences in ASE

Independent-samples *t* tests showed group differences in ASE by gender, grade, and class officer status (Table 1). Boys scored higher than girls on academic competence self-efficacy and overall ASE. Seventh graders scored higher than eighth graders on overall ASE and on all dimensions. Class officers also reported higher ASE (overall and by dimension) than non-officers.

#### 3.2.2. Associations Among PTS, Academic Emotions, and ASE

Pearson correlations indicated that PTS was significantly related to PAE, NAE, academic competence self-efficacy, and academic behavior self-efficacy (Table 2).

### 3.3. Mediating Analyses

#### 3.3.1. Parallel Mediation via PAE and NAE

**Figure 2:**
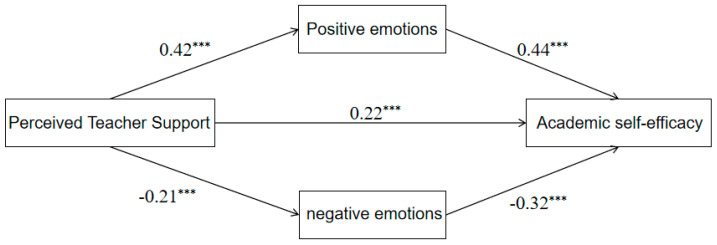
The Predictive Role of PTS on ASE: The Concurrent Mediating Role of Academic Emotion, *** *p* < 0.001.

Given the significant influence of demographic variables on ASE, this study incorporates gender, grade and class officers as covariates in all mediation models to enhance the robustness of the estimates. Parallel mediation was tested with PROCESS (Model 4) (Hayes, 2013). Both indirect paths—PAE (*β* = 0.42, *t* = 8.77, *p* < 0.001) and NAE (*β* = −0.21, *t* = −4.08, *p* < 0.001) (Table 3)—were significant, and the parallel mediating effect of PAE and NAE was established. The specific mediation model is shown in Figure 2.

To assess the significance of the mediating effect, we employed the bias-corrected Bootstrap method (Fang & Zhang, 2012). The analysis results (see Table 4) indicate that the Bootstrap 95% confidence intervals for all pathways exclude zero, confirming the mediating effect. Furthermore, the difference in indirect effects between positive and negative academic emotions was 0.12 (Bootstrap SE = 0.03), with its Bootstrap 95% confidence interval also excluding zero. This finding demonstrates that the mediating effect of PAE is significantly stronger than that of NAE.

#### 3.3.2. Mediation by ASE Dimensions

**Figure 3:**
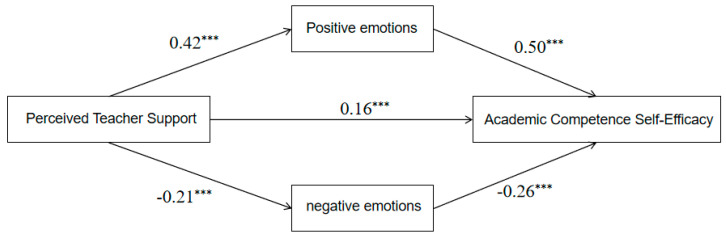
The Predictive Effect of PTS on Academic Competence Self-Efficacy: The Concurrent Mediating Role of Academic Emotion, *** *p* < 0.001.

**Figure 4:**
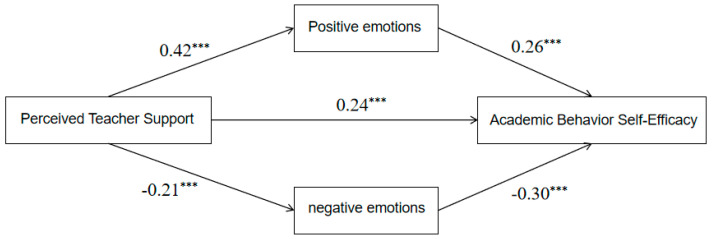
The Predictive Effect of PTS on Academic Behavior Self-Efficacy: The Concurrent Mediating Role of Academic Emotion, *** *p* < 0.001.

Two parallel mediation models were estimated separately with academic competence self-efficacy (Model 1) and academic behavior self-efficacy (Model 2) as outcomes, and PAE/NAE as parallel mediators; gender, grade, and class officer were also included as covariates in the model. PTS significantly predicted academic competence self-efficacy (*β* = 0.17, *t* = 4.72, *p* < 0.001) and academic behavior self-efficacy (*β* = 0.24, *t* = 5.16, *p* < 0.001) through the emotion pathways (Table 5). The mediation effect analysis and paired comparison analysis revealed that in the academic competence self-efficacy model, the difference in mediation effects between positive and negative academic emotions was 0.09 (Bootstrap SE = 0.02), with its 95% confidence interval excluding zero. This indicates that in predicting academic competence self-efficacy through PTS, the mediation effect of PAE is significantly stronger than that of NAE. In the academic behavior self-efficacy model, the difference in mediation effects between positive and negative academic emotions was 0.02 (Bootstrap SE = 0.02), with its 95% confidence interval, including zero. This result suggests that there is no significant difference in the mediation effects between PAE and NAE. Full path coefficients are reported in Table 6, with diagrams shown in Figure 3 and Figure 4.

## 4. Discussion

This study took students from a junior high school in central China as samples to examine the relationship between PTS and ASE, and tested the parallel mediating role of PAE and NAE. The results showed that PTS had a significant positive predictive effect on junior high school students’ ASE; PAE and NAE played a significant parallel mediating role between the two; when predicting different dimensions of ASE, the mediating effect strength of the two types of emotions showed differences.

### 4.1. Differences in ASE Among Junior High School Students

Clear gender differences emerged in ASE, with the largest gap observed in academic competence self-efficacy, where girls scored lower than boys. These differences can be explained from the perspective of the integration of social cognitive theory and social role theory. This pattern aligns with prior evidence (Hanham et al., 2021; Kim et al., 2025). Syntheses of recent work suggest that gender stereotyping, subject-linked positioning, and broader social expectations can weaken girls’ beliefs about academic capability (L. H. Wang & Yu, 2023). Social role theory further explains this tendency by arguing that normative role expectations are internalized and incorporated into self-evaluations (Koenig & Eagly, 2014). Female students are often expected to be diligent and serious, which may lead them to attribute success more readily to effort and to adopt a more conservative attributional style regarding ability. Meanwhile, Previous studies have indicated that in fields perceived as male-dominated (e.g., some STEM areas), female students may experience additional psychological burden and anxiety due to fears of confirming negative stereotypes. This can undermine their self-efficacy beliefs and academic performance (Ho et al., 2025; Shapiro & Williams, 2012). Conversely, previous studies have indicated that male students benefit from the stereotype that they are innately suited for STEM fields, which suggests that boys are more likely to interpret setbacks as resulting from external or unstable causes, thereby helping to preserve higher levels of academic competence self-efficacy (Cuder et al., 2024; L. H. Wang & Yu, 2023). The findings suggest that the gender differences are not caused by the differences in ability itself, but by the interaction of attribution patterns and emotional experiences in the social cognition process.

With respect to grade differences, seventh graders scored significantly higher than eighth graders on ASE, both for the overall score and for each dimension. This pattern likely reflects the combined effects of psychological transitions during adolescence and increasing subject difficulty, consistent with prior research showing declines in academic self-efficacy during mid-adolescence or periods of heightened academic demands (Liao & Zhang, 2023). More specifically, eighth-grade students are at a key stage of cognitive maturation and self-awareness development, and enhanced critical thinking may enable them to evaluate their strengths and limitations more analytically. At the same time, curricular difficulty increases markedly in eighth grade (e.g., introduction of physics and geometry). Under these dual pressures, students may be more inclined to attribute academic difficulties to stable internal causes, such as perceived lack of ability (Travis et al., 2020). According to social cognitive theory, such maladaptive attribution patterns can directly weaken ASE, which may help explain the commonly observed decline at this stage (Bandura, 1997).

Furthermore, students who served as class officers exhibited significantly higher academic self-efficacy than students without such roles. Social cognitive theory provides a robust explanation for this phenomenon (Bandura, 1997). First, holding a class-officer role provides students with a positive leader identity. This identity motivates students to align their behavior with role expectations, leading to greater responsibility and perceived competence (Hirosawa et al., 2024; Stryker & Burke, 2000). Class-officer students may hold an advantage for two reasons. They often receive more teacher attention, feedback, and emotional affirmation, which corresponds to verbal persuasion in Bandura’s sources of efficacy. Their role performance also creates repeated mastery opportunities, a particularly potent basis for efficacy development (Asrori & Tjalla, 2024). Together, role-related success and sustained support may reinforce ASE via competence validation and accumulated successful experiences.

### 4.2. The Direct Predictive Effect of PTS on ASE

PTS showed a robust positive link with junior high students’ ASE, consistent with Sun et al. (2021). This finding supports the view that PTS functions as an important contextual resource for efficacy formation. From the perspective of social cognitive theory, self-efficacy is formed through the interaction between individuals and their environment. Among the four major sources of efficacy information, persuasive speech from significant others plays a particularly crucial role during adolescence (Usher & Schunk, 2017). Moreover, the predictive effect of perceived teacher support on academic self-efficacy is consistent with the findings of domestic scholars based on samples from other regions (C. Wang et al., 2024; Zhou et al., 2025; Chai et al., 2011), indicating that this mechanism exhibits certain cross-regional stability in the context of China. Learning, emotional, and capability support are each positively related to ASE and its facets (Chai et al., 2011). Learning support (e.g., strategy instruction) can directly raise students’ confidence in handling complex academic tasks by offering clear guidance and modeling effective approaches (Y. X. Wang et al., 2024). Emotional support (e.g., caring encouragement) helps create a positive and secure classroom climate, which provides an affective basis for students to confront academic challenges (Zhou et al., 2025). Capability support (e.g., providing meaningful choice) may facilitate mastery experiences through appropriately challenging tasks and timely feedback, thereby consolidating ASE (Bandura, 1997).

It is worth emphasizing that the teacher expectation effect can be regarded as a further elaboration of “verbal persuasion” in social cognitive theory: teachers can subtly influence students’ self-perception and learning behaviors by conveying high expectations, providing differentiated feedback, and assigning challenging tasks (Rosenthal & Jacobson, 1968). This process manifested as teachers’ supportive behaviors conveying positive expectations (e.g., “You can do it”), thereby encouraging students to develop corresponding academic self-efficacy (Bronfenbrenner & Evans, 2000). For instance, a longitudinal study of first-grade students in Germany found that teacher support promoted learning development via classroom feedback, offering empirical support for expectancy-based mechanisms (Ruberdavies & Hattie, 2025). In conclusion, this study demonstrates from both empirical and theoretical perspectives that PTS, through expectation transmission and supportive environment creation, jointly promotes the development of ASE in junior high school students across three dimensions: learning, competence, and emotional support. This finding is highly consistent with existing theoretical explanations.

### 4.3. Parallel Mediation via Academic Emotions

This study confirms that PAE and NAE play a significant parallel mediating role between PTS and ASE. PAE and NAE operated as concurrent mediators between PTS and ASE. Unlike single-route accounts that prioritize one emotional pathway (An et al., 2023), the present model captures a two-channel process: PTS can enhance ASE by improving PAE, and can weaken the negative effect of NAE on ASE by reducing NAE. This study builds upon previous research. Specifically, while Lei et al. (2018) established a positive correlation between teacher support and positive academic emotions, our research further reveals that the elevation of these emotions serves as a critical intermediary in transforming teacher support into students ‘academic confidence. Additionally, the negative academic emotion pathway confirmed in this study aligns with the findings of McGugan et al. (2023): supportive teacher interactions effectively alleviate learners ‘negative emotional experiences. Through a parallel mediation model, our study demonstrates that buffering negative academic emotions ultimately enhances students’ ASE. This finding addresses the limitations of previous research that focused on “single-emotion dominance” (Chen et al., 2022; Villavicencio & Bernardo, 2016; Zhao & Liu, 2022), and suggests that educational interventions should both cultivate positive emotions and manage negative emotions.

Along the positive pathway, PTS was linked to higher ASE via increased PAE (e.g., enjoyment, excitement, satisfaction, and pride) (Schunk & Dibenedetto, 2020). This finding aligns with the core tenets of the control-value theory: teacher support enhances students’ sense of control and value in learning activities, thereby fostering positive emotions (Pekrun, 2006). Meanwhile, the Broaden-and-Build Theory further elucidates the functions of positive emotions (Fredrickson, 2001): Positive affect can broaden momentary cognitive–behavioral repertoires and encourage deeper learning strategies. Over time, these benefits may accumulate into more durable psychological and cognitive resources. Such resources can increase opportunities for mastery experiences and sustained coping with academic demands. Empirically, Ren et al. (2022) reported a chain-mediated link in which positive emotions and achievement goals connected teacher support to self-efficacy. Related evidence from domestic studies likewise suggests an indirect association between teacher support and ASE through positive emotions (Chai et al., 2011).

On the mediating path of NAE, teacher support may reduce anxiety, boredom, and fatigue, thereby safeguarding ASE. This interpretation is consistent with control–value theory, which locates academic emotions in students’ appraisals of control (e.g., competence, autonomy) and value (e.g., importance, enjoyment) (Pekrun, 2024). Supportive practices can strengthen these appraisals and, in turn, curb negative emotional experiences. Teachers can raise perceived control by offering strategic support and meaningful autonomy opportunities. They can also enhance perceived value through emotional care and clear explanations of task meaning. These appraisals can, in turn, reduce anxiety and boredom in learning contexts. When negative emotions decline, cognitive resources and motivation may be released rather than suppressed. Granziera et al. (2022) found that teachers’ emotional support was negatively related to students’ anxiety and boredom. Conversely, negative teacher–student interactions (e.g., indifference or blame) have been linked to stronger negative emotions and weaker motivation and self-concept (Brandmiller et al., 2020; Feng & Xiao, 2024; Y. X. Wang et al., 2024). The above empirical research collectively provides support for the perception that PTS reduces NAE and is then correlated with ASE.

The two emotional pathways showed different strengths across dimensions of ASE. For academic competence self-efficacy, the indirect effect via positive emotions was notably larger than the indirect effect via negative emotions. This suggests that teacher support may build confidence in knowledge mastery and cognitive growth primarily by energizing positive feelings. Such an interpretation fits the long-term constructive role of positive emotions emphasized by broaden-and-build theory (Fredrickson, 2001). Consistent evidence also shows a particularly strong mediating role for positive emotions in the teacher support–self-efficacy link (C. Wang et al., 2024).

For behavior-related self-efficacy, the indirect effects through positive and negative emotions were similar in magnitude. Self-efficacy in academic behaviors refers to students’ confidence in specific learning actions such as task initiation and time management. Negative emotions—like anxiety-induced procrastination—exert an immediate and direct disruptive effect on these behaviors. Although positive emotions can temporarily boost behavioral motivation, they struggle to directly establish stable behavioral routines and habits. Therefore, at the behavioral level, the “constructive” advantage of positive emotions diminishes while the “destructive” effect of negative emotions becomes more pronounced, leading to a convergence in the strength of influence between the two pathways. Recent work likewise suggests that teacher support relates to self-monitoring both by boosting positive emotions and by regulating negative emotions (Sadoughi & Hejazi, 2021).

Bandura’s self-efficacy theory offers a coherent explanation for this pattern (Bandura, 1997). Academic competence self-efficacy relies more on cognitive depth and mastery experiences, and it may therefore be more responsive to positive emotions. In contrast, behavior-related self-efficacy depends on sustained action, which requires both motivational energy and reduced emotional interference. Accordingly, both positive and negative emotions are meaningfully involved in shaping behavioral self-efficacy. Overall, the evidence favors a dual-channel account: PTS promotes ASE through emotional activation (positive affect) and emotional buffering (reduced NAE). The dimension-level comparison further refines this mechanism by showing that these routes can differ in relative influence across self-efficacy facets.

In summary, this study deepens existing understanding from both theoretical and situational perspectives. On one hand, the research findings align closely with social cognitive theory (Bandura, 1997) and control-value theory (Pekrun, 2006), validating the dual-channel mechanism whereby PTS can directly predict ASE or be mediated by academic emotions to predict it. On the other hand, the study extends the theoretical framework, primarily derived from Western higher education, to the Chinese junior high school population, providing empirical support for its applicability in Eastern culture and basic education. Simultaneously, this research reveals the dual-channel model of emotions—the activation channel for PAE and the buffering channel for NAE—offering a more comprehensive theoretical framework than a single emotional pathway for understanding the mechanisms of teacher support. It also refines the dimensions of ASE, discovering that the two types of emotions exhibit differentiated patterns in predicting ability, self-efficacy and behavioral self-efficacy, providing a new perspective for future research on structural differences in ASE and its relationship with emotions.

### 4.4. Research Limitations and Prospects

This study has the following limitations: First, it adopts a cross-sectional design, with all variables measured at the same time point, making it difficult to infer causal relationships between variables. Even though this study tested the mediating path based on theoretical hypotheses, “mediation” here is a statistical concept referring to the existence of indirect associations rather than the confirmation of causal chains. Future research could adopt a longitudinal study design and combine multilevel modeling at the class or school level to more rigorously explore the changing paths of ASE and its formation mechanisms from a dynamic developmental perspective. Second, the data primarily come from student self-reports, which may carry the risk of common method bias. Although this study used established scales and standardized administration procedures and conducted relevant statistical tests to control the impact of this bias to some extent, a single data source may still amplify the relationships between variables. Future research could introduce classroom observations, video analysis, and reports from multiple stakeholders, such as teachers and parents, to form more comprehensive multi-source data and enhance the robustness of conclusions. Finally, in terms of sample representativeness, the sample size of this study is relatively limited, and the data are only sourced from a junior high school in a city in central China. It should be noted that the selection of this school as the research object was mainly based on the feasibility of the study implementation. Compared to the developed eastern regions, the school is located in an area with moderate economic development and moderate educational standards, making it somewhat representative of the local context and able to reflect the general situation of junior high schools in grassroots China. This design helps control for irrelevant variables at the school level in a relatively homogeneous context, thereby enhancing the internal validity of the study. However, the single-source sample still significantly limits the generalizability of the findings to other regions (e.g., eastern and western areas) or other types of schools (e.g., high schools). Therefore, this study is more of an evidence supplement to the “mechanism of ASE formation among junior high school students in China’s urban grassroots,” rather than an unconditional inference for all schools nationwide. Future research could expand the sample size across different regions and school types, systematically describe school and regional characteristics, and test the potential moderating effects of situational factors on model pathways, thereby providing a more comprehensive understanding of the development patterns of ASE.

### 4.5. Educational Implications

#### 4.5.1. Foster a Highly Supportive Classroom Environment to Solidify the Developmental Foundation for Self-Efficacy

This study suggests that a highly supportive classroom environment may serve as a crucial external condition for fostering students ‘ASE. Educators are advised to provide support across three dimensions: learning, competence, and emotion. Learning support can be implemented through “teaching how to fish” by offering strategic guidance, resources, and formative feedback to help students accumulate mastery experiences. Competence support should focus on instilling growth mindset beliefs, utilizing growth-oriented feedback that emphasizes the development process to enhance students’ awareness of their potential. Emotional support primarily involves creating a safe and trusting environment to strengthen students’ sense of belonging. The synergistic effects of these three dimensions are expected to lay a solid foundation for the development of ASE.

#### 4.5.2. Integrate Emotion Awareness and Regulation into Instruction to Build a Synergy Between Emotion and Cognition

Integrating emotional cognition and regulation into daily teaching serves as a potential preventive strategy to enhance ASE. Educators may adopt a “dual-track approach”: On one hand, they can stimulate and reinforce positive emotions by establishing clear triggers, strengthening learning control and value experiences, and cultivating joy and pride. On the other hand, they can guide students to scientifically manage emotional fluctuations, apply coping techniques, and attempt to break the vicious cycle between negative emotions and low efficacy through cognitive reappraisal strategies. By systematically developing skills in emotion recognition, expression, and regulation, students’ emotional autonomy can be strengthened, laying a stable internal foundation for the sustained growth of ASE.

#### 4.5.3. Implement Differentiated Interventions Targeting ASE Dimensions to Achieve Targeted Support

Based on the findings of this study, the formation mechanisms of academic competence self-efficacy (likely primarily driven by positive academic emotions) and academic behavioral self-efficacy (potentially influenced by both positive emotional reinforcement and negative emotional mitigation) differ. Therefore, targeted dimensional interventions can be implemented. For academic competence self-efficacy, it is recommended to establish a belief system grounded in positive emotions: By designing “micro-success” experiences to accumulate mastery, reinforcing positive emotions and growth-oriented attribution, and solidifying the belief that “abilities can be enhanced.” For academic behavioral self-efficacy, dual support through emotional regulation and behavioral scaffolding can be attempted: Utilizing PAE to boost pleasure and persistence, while transforming NAE and supplementing with behavioral management strategies (e.g., goal restructuring, plan execution monitoring) to convert stress into actionable behaviors. These two types of interventions are interconnected. If synergized, they may help teachers transition from “one-size-fits-all teaching” to “need-based support,” thereby effectively enhancing students’ overall ASE. It should be noted that the above findings are preliminary discoveries from a specific sample in this study, and their generalizability requires further validation in future research within broader educational contexts.

## 5. Conclusions

Taken together, the present findings clarify how teacher-related support is linked to junior high school students’ ASE. PTS was positively associated with ASE, suggesting that greater PTS corresponded to stronger student confidence in addressing academic demands. This association operated through two routes: a direct link from PTS to ASE and indirect links carried by students’ emotional experiences, including PAE and NAE. In other words, supportive teacher behaviors strengthen students’ efficacy beliefs not only by providing immediate encouragement and guidance but also by shaping the emotional tone of learning. When ASE was examined by dimension, the indirect pathway via PAE was comparatively stronger for academic competence self-efficacy. By contrast, for academic behavior self-efficacy, the indirect effects via PAE and NAE were similar in magnitude. Overall, these results suggest that enhancing students’ PAE may be particularly important for strengthening academic competence self-efficacy, whereas both emotional channels appear relevant to academic behavior self-efficacy.

## Figures and Tables

**Table 1:** Analysis of Differences in Academic Self-Efficacy.

Project	Variable	Academic Competence Self-Efficacy		Academic Behavior Self-Efficacy		Academic Self-Efficacy	
		*M ± SD*	*t*	*M ± SD*	*t*	*M ± SD*	*t*
Gender	Male	40.31 ± 8.36	3.66 ***	38.40 ± 6.88	0.37	78.71 ± 13.56	2.42 *
	Female	37.19 ± 8.20		38.14 ± 6.85		75.32 ± 13.55	
Grade	Grade 7	39.85 ± 8.44	3.03 **	38.90 ± 7.16	2.13 *	78.75 ± 13.92	2.94 **
	Grade 8	37.21 ± 8.16		37.38 ± 6.34		74.58 ± 12.89	
Class officers	Yes	40.29 ± 8.33	3.53 ***	39.07 ± 6.68	2.25 *	79.36 ± 13.43	3.31 ***
	No	37.25 ± 8.29		37.48 ± 6.98		74.73 ± 13.55	

* *p* < 0.05, ** *p* < 0.01, *** *p* < 0.001.

**Table 2:** Correlation Analysis Results for Each Variable (n = 376).

	*M*	*SD*	1.	2.	3.	4.	5.	6.
1. Perceived teacher support	84.35	15.14	—					
2. Academic self-efficacy	77.02	13.64	0.49 **	—				
3. Academic competence self-efficacy	38.75	8.41	0.46 **	0.91 **	—			
4. Academic behavior self-efficacy	38.27	6.86	0.42 **	0.87 **	0.59 **	—		
5. Positive academic emotion	111.91	14.77	0.44 **	0.71 **	0.74 **	0.52 **	—	
6. Negative academic emotion	116.34	27.16	−0.24 **	−0.61 **	−0.60 **	−0.49 **	−0.54 **	—

** *p* < 0.01.

**Table 3:** Regression Analysis of PTS, Academic Emotion, and ASE.

Result Variable	Predictor Variable	*R*	*R* ^2^	*F*	*β*	*t*
Academic self-efficacy	Perceived teacher support	0.53	0.28	35.63 ***	0.47	10.22 ***
Positive academic emotion	Perceived teacher support	0.47	0.22	25.67 ***	0.42	8.77 ***
Negative academic emotion	Perceived teacher support	0.32	0.11	10.94 ***	−0.21	−4.08 ***
Academic self-efficacy	Perceived teacher support	0.79	0.63	104.66 ***	0.22	6.07 ***
	Positive academic emotion				0.44	10.68 ***
	Negative academic emotion				−0.32	−8.23 ***

Note: This regression model controls for three categorical covariates—gender, grade level, and class officer—all of which were not standardized, hence the unstandardized regression coefficients B are not presented in the table. All core predictive variables were standardized, and the standardized regression coefficients *β* are displayed in the table, *** *p* < 0.001.

**Table 4:** Mediating Effect Analysis of Academic Emotion.

	Mediating Variable	Effect	Boot SE	Bootstrap 95%	
				Boot LLCI	Boot ULCI
Total Effect	/	0.47	0.05	0.38	0.56
Direct Effect	/	0.22	0.04	0.15	0.29
Indirect Effect	Positive academic emotion	0.18	0.03	0.13	0.23
	Negative academic emotion	0.07	0.02	0.03	0.10
Contrast	Positive vs. Negative	0.12	0.03	0.06	0.17

**Table 5:** Regression Analysis of PTS on Dimensions of ASE.

Result Variable	Predictor Variable	*R*	*R* ^2^	*F*	*β*	*t*
Model 1: Academic competence self-efficacy						
Academic competence self-efficacy	Perceived teacher support	0.51	0.27	33.93 ***	0.42	9.16 ***
Positive academic emotion	Perceived teacher support	0.47	0.22	25.67 ***	0.42	8.77 ***
Negative academic emotion	Perceived teacher support	0.32	0.11	10.94 ***	−0.21	−4.08 ***
Academic competence self-efficacy	Perceived teacher support	0.80	0.63	106.71 ***	0.16	4.40 ***
	Positive academic emotion				0.50	12.28 ***
	Negative academic emotion				−0.26	−6.93 ***
Model 2: Academic behavior self-efficacy						
Academic behavior self-efficacy	Perceived teacher support	0.43	0.19	21.06 ***	0.41	8.48 ***
Positive academic emotion	Perceived teacher support	0.47	0.22	25.67 ***	0.42	8.77 ***
Negative academic emotion	Perceived teacher support	0.32	0.11	10.94 ***	−0.21	−4.08 ***
Academic behavior self-efficacy	Perceived teacher support	0.62	0.38	38.20 ***	0.24	5.19 ***
	Positive academic emotion				0.26	4.85 ***
	Negative academic emotion				−0.30	−6.14 ***

Note: This regression model controls for three categorical covariates—gender, grade level, and class officer—all of which were not standardized, hence the unstandardized regression coefficients B are not presented in the table. All core predictive variables were standardized, and the standardized regression coefficients *β* are displayed in the table, *** *p* < 0.001.

**Table 6:** Analysis of the Mediating Effects of PTS on Dimensions of ASE.

	Mediating Variable	Effect	Boot SE	Bootstrap 95%	
				Boot LLCL	Boot ULCL
Model 1 Academic competence self-efficacy					
Total Effect	/	0.42	0.05	0.33	0.51
Direct Effect	/	0.16	0.04	0.09	0.23
Indirect Effect	Positive academic emotion	0.21	0.03	0.15	0.27
	Negative academic emotion	0.05	0.02	0.03	0.09
Contrast	Positive vs. Negative	0.15	0.03	0.09	0.22
Model 2: Academic behavior self-efficacy					
Total Effect	/	0.41	0.05	0.32	0.51
Direct Effect	/	0.24	0.05	0.15	0.33
Indirect Effect	Positive academic emotion	0.11	0.03	0.06	0.16
	Negative academic emotion	0.06	0.02	0.03	0.10
Contrast	Positive vs. Negative	0.04	0.03	−0.02	0.11

## Data Availability

The datasets used and/or analysed during the current study are available from the corresponding author on reasonable request.

## Abbreviations

The following abbreviations are used in this manuscript:

ASE	Academic self-efficacy
PTS	Perceived teacher support
NAE	Negative academic emotions
PAE	Positive academic emotions
